# Widespread Suspected Illegal Advertising of Paid Loot Boxes and Social Casino Games in Belgium: Empowered by the EU Digital Services Act to Assess Compliance Using Meta’s Ad Repository

**DOI:** 10.1159/ear/acrag008

**Published:** 2026-10-17

**Authors:** Leon Y. Xiao, Elena Petrovskaya, Nicole Khoo, Maarten Denoo, Amanda Roberts

**Affiliations:** a School of Creative Media, City University of Hong Kong, Hong Kong, Hong Kong SAR; b beClaws.org, London, UK; c College of Health and Science, University of Lincoln, Lincoln, UK; d Department of Computer Science and Technology, University of Cambridge, Cambridge, UK; e School of Computer Science and Digital Technologies, London South Bank University, London, UK; f Media Culture and Policy Lab, KU Leuven, Belgium; g School of Sport, Exercise and Health Sciences, Loughborough University, Loughborough, UK

**Keywords:** Loot boxes, Gacha, Video games law, Videogaming regulation, Gambling, Advertising regulation, Consumer protection, Belgium, EU Digital Services Act, Transparency, Social media

## Abstract

**Introduction:**

Paid loot boxes and social casino mechanics are gambling-like products inside video games that provide random rewards in exchange for real-world money. These products may cause harm to vulnerable consumers, e.g., children and adults experiencing gambling harms. Most countries do not presently regulate these mechanics as gambling because the random rewards do not possess real-world monetary value as defined by law. Uniquely, Belgium’s gambling law does not have that requirement, where paid loot boxes and social casinos therefore constitute unlicensed and illegal gambling, and providing these products is a criminal offence. However, this “ban” has not been enforced due to the gambling regulator lacking resources, so these products remain widely available. We investigated whether, beyond failing to remove these products from the Belgian version of app stores, companies are also paying to advertise games containing them on social media to specifically attract Belgian users, which would constitute an additional crime of advertising illegal gambling.

**Methods:**

We gained access to demographic-based (country, age, and gender) targeting and reach data through the EU Digital Services Act, which requires very large online platforms (e.g., Instagram and Facebook) to provide repositories for all advertising shown and associated audience demographic data.

**Results:**

Concerningly, 172 popular games with illegal paid loot boxes and social casino games were widely advertised in Belgium. We studied 1,574 ads, which were viewed over 4.5 million times by Belgian users, including conceivably 544,000 times by under-21s (estimated based on our hypothetical assumptions), who are not legally permitted to gamble online in Belgium.

**Conclusion:**

Policymakers should hold social media platforms and app stores accountable for conducting effective content moderation and ensuring illegal games and ads are not available to local users. More data access opportunities should be mandated by law in the EU and beyond to aid research and policy enforcement.

## Introduction

Video games are increasingly monetized by in-game purchases (where players are invited to buy additional items and services inside the game with real-world money), particularly on mobile phone platforms, such as iOS and Android. One type of such transactions has been a particular cause of concern for players, parents, and policymakers: “paid loot boxes” [1–3]. Players buy these gambling-like products with real money to obtain random rewards. Around 80% of the highest grossing games in Western countries [4–8] and more than 90% in East Asian countries sell these gambling-like in-game purchases [9–11]. Paid loot boxes are often available to children because many games selling them are given age ratings signifying their supposed suitability for young people, including children as young as four years old in many cases [12].

Paid loot boxes are recognized to share psychological and structural similarities with traditional gambling [13, 14] and similar risk factors as other addictive products, e.g., drugs and alcohol [15]. Research has consistently found a link between monetary spending on paid loot boxes and harmful gambling symptomology [16–19]. Longitudinally, young people who purchased paid loot boxes were more likely to participate in and spend more money on traditional gambling six months later [20–22]. Consequently, paid loot boxes have attracted significant academic and regulatory scrutiny.

Another related product, arguably of even more concern than paid loot boxes, is the “social casino” category of video games [23, 24]. Social casino games allow players to spend real-world money online to purchase virtual currency to “simulate” participating in traditional gambling activities, such as using slot machines and playing blackjack and poker [25, 26]. Importantly, the player has no opportunity to convert any winnings of virtual currency back into real-world money. Nonetheless, consumers can lose significant amounts of money and experience significant harms from engaging with these games [24, 27, 28]. Social casino games frequently glamorize traditional gambling [29] and are often much more aesthetically and mechanically similar to traditional gambling than most paid loot boxes.

### Belgium as a Case Study for Prohibitionist Regulation

Countries around the world regulate paid loot boxes and social casino games differently with varying degrees of restriction [30–32], and those regulations change rapidly [33]. For example, some countries require that specific information be disclosed, such as the presence of paid loot boxes on physical packaging [34], app store product listings [35, 36], and any advertising [37–39]. The probability of obtaining different rewards must also be provided in some countries [4, 9]. As to social casino games in particular, these are generally not regulated above and beyond any other video game, although Australian law was recently changed to require them to be legally restricted to adults only (18+) from September 2024 [40, 41]. Importantly, the commonality is that most countries do not regulate most paid loot boxes and any social casino games as a form of “gambling” because with few exceptions, the player can never win real-world money [30–32].

Belgium is an exception to this rule: its broadly defined gambling law means that all in-game purchases involving randomization are regulated as illegal gambling [5, 30], thus covering both paid loot boxes and social casino games. In 2015, the highest administrative court in Belgium ruled that a social casino game available on a website (as compared to one found inside its own mobile app) satisfies the legal definition of gambling and was illegal when offered without a license (Paragraph 13 of *GamePoint v Belgium*) [42]. Similarly, in 2018, the Belgian gambling regulator publicly opined that paid loot boxes constitute illegal gambling and threatened criminal prosecution [43]. This interpretation that paid loot boxes breach Belgian gambling law has since been confirmed by the Enterprise Court of Antwerp in January 2025 (Paragraph 10 of *LS v Apple*) [44, 45]. Video games that sell paid loot boxes and social casino games are therefore prohibited, and companies risk sanctions by offering them in Belgium.

Despite this supposed prohibition, previous research in Belgium has found that 82 of the 100 highest grossing iPhone games continued to sell paid loot boxes illegally and/or were illegal social casino games [5]. A few companies have taken compliance action by either removing paid loot boxes from the Belgian version of the game or withdrawing their games with paid loot boxes from the Belgian market entirely [46]. However, players are able to easily circumvent those rarely applied restrictions using various methods, such as by using a virtual private network (VPN) to make their internet protocol address appear to not be from Belgium or simply changing their device’s app store country setting to a different country (e.g., the Netherlands or France) [5]. At least 13.3% of Flemish (i.e., Dutch-speaking Belgian) teenagers between 11 and 18 who open paid loot boxes explicitly admitted to having done so by circumventing restrictions [47]. The Belgian gambling regulator [48] and the then Minister of Justice [49] have admitted in response to academic research [5] that the ban is difficult to enforce and may not be proportionate, such that law reform and other regulatory options should be considered, including even lifting the ban and permitting paid loot boxes that comply with consumer protection law in the future [50].

While it is known that many companies are culpably failing to effectively make an illegal product unavailable to Belgian consumers, a hitherto unexplored aspect is whether companies are actively paying to advertise video games with paid loot boxes to attract Belgian consumers. The link between advertising and traditional gambling-related harm is well established [51–54]. Advertising illegal gambling would entice more consumers into engagement and spending money, which would constitute a separate criminal offence of promoting illegal gambling (Article 4 § 2 of the Belgian Gambling Act of 7 May 1999), punishable by a fine of up to EUR 576,000 (Article 64). Young people aged under 21 years are prohibited from participating in online gambling (except any National Lottery products, which are irrelevant for present purposes) even when the activity has been appropriately licensed by the Belgian gambling regulator (Article 54 § 1). For that reason, the criminal penalty for advertising illegal unlicensed paid loot boxes and social casino games may be doubled to a fine of up to EUR 1.152 million if the offence is committed against a person aged under 21 years (Article 65), i.e., if the social media ads targeted and/or reached under-21s.

The advertising of paid loot boxes and social casino mechanics specifically is certainly prohibited under Belgian criminal law. We further argue that the advertising of the overarching video game containing paid loot boxes and social casino mechanics would also be prohibited (or at least be highly reproachable). This would be true regardless of the ad’s content, including even when neither illegal gambling product is directly referenced in the ad: for example, when only other aspects (e.g., gameplay and storyline) were advertised. The presence of illegal gambling in a video game renders that whole video game an illegal product that should not be advertised to the public. These games are being advertised because the companies want consumers to download them and spend money on them. Considering that many games, particularly on mobile platforms, are highly, if not wholly, reliant on paid loot boxes and social casino mechanics to generate revenue, an advertisement to download and play the game, even when it does not necessarily highlight those mechanics, would always be an attempt by the company to convince players to ultimately spend money on those very mechanics inside that game, thereby promoting illegal gambling. Notably, social casino games in particular would be highly difficult to advertise without referencing the social casino mechanics, as those games would have little else to offer.

### Studying Social Media Advertising Using Mandated Data Repositories

It is only very recently that the ability to systematically investigate social media advertising became available [37]. Article 39 of the recently adopted EU Digital Services Act (DSA) requires leading social media platforms to provide a repository of all advertising shown in EU countries (including Belgium) and publicly disclose, inter alia, whether Belgian users were targeted or excluded from potentially being shown the advert; which demographics were targeted; and how many Belgian users belonging to each demographic group (e.g., young men aged between 25 and 34) in fact saw the advert. For example, the Meta Ad Library is a repository of all paid advertising shown on Meta-owned social media platforms (e.g., Facebook and Instagram). As required by the DSA, this database provides a complete and accurate reproduction of the original ad (including any accompanying text and static images or video) and associated data on, e.g., when the ad was shown; what age groups and genders it targeted; and how many people in various demographic groups saw it.

To inform the public and evidence-based enforcement and policymaking, it is important to assess the implementation of existing regulations, particularly in relation to novel technologies. If those policies are not effective in practice, then they ought to be more strongly enforced or reconsidered for repeal, so that the public is not misled as to whether they are sufficiently protected by existing law [5]. Through the present study, we sought to understand whether and, if so, to what extent companies are deliberately advertising popular video games containing gambling-like mechanics (i.e., paid loot boxes and social casino games) that constitute illegal gambling to Belgian users on social media.

We are able to distinguish between the paid loot boxes and social casino games because whether a game predominantly constitutes a social casino game (e.g., by featuring traditional gambling motifs and activities) is an assessment that could be made based on objective criteria [5]. We specifically highlight the compliance situation among social casino games because they are perceived as more concerning than other forms of in-game purchases involving randomization [23, 24], e.g., paid loot boxes and “gacha” mechanics that are often found in games produced in East Asia that also offer random rewards in exchange for money but are not aesthetically represented as a box or treasure chest being opened [55, 56]. The research motivations, questions, and methods were preregistered in the Open Science Framework at https://doi.org/10.17605/OSF.IO/AJM7F.

## Methods

A list of 390 video game titles known to contain paid loot boxes and/or constitute social casino games was generated using data from prior research [4, 5, 9, 10, 12, 35, 36]. In addition, five games (*WWE 2K24*, *NBA 2K25*, *EA SPORTS FC 24*, *EA SPORTS FC 25*, and *EA SPORTS FC 25 Companion*) were added to the list as they are part of game series that are released annually and were the then newest rendition of other games already in the list (e.g., *WWE 2K23*) that were no longer advertised due to having been effectively replaced by the newer games. During data collection, 23 entries were found to have been a duplicate of another entry and were therefore excluded, leaving 372 unique game titles as shown in online supplementary material 1 (for all online suppl. material, available at https://doi.org/10.1159/ear/acrag008), which is a full list of those titles showing whether they advertised in Belgium and/or the Netherlands.

All games were separately searched for using the Meta Ad Library (https://www.facebook.com/ads/library) with the country option set to Belgium and the Netherlands to check whether they advertised in those two countries. For the games that advertised, a list of all recent ads would have been displayed. We chose the Netherlands as a comparator because it shares geographic and cultural proximity to Belgium but did not have any active regulations against paid loot boxes, social casino games, or their advertising during the data collection period [57].

Data were collected between October 2024 and February 2025. Up to 10 of the most recent ads for each game title shown in Belgium were sampled. Besides the graphics, based on the data provided by the repository, we recorded demographic-related information about whom the ad was intended to target (i.e., which genders and what age range) and whom it eventually reached (i.e., user impressions).

### Inter-Rater Reliability

A random list of 40 games was assessed by a second coder as to whether those games advertised in Belgium and the Netherlands. In the first pass, for Belgium, this established a Krippendorff’s alpha of 0.79, which was on the border of a satisfactory level [58, 59]. For the Netherlands, Krippendorff’s alpha was 0.75. The discrepancies were discussed within the team, and it was found that many were due to different expectations between coders as to how the ads should be searched (e.g., what search terms based on the game’s title should be used). With all the discrepancies resolved, a further random list of 40 games was double coded by the same second coder. The second pass established a Krippendorff’s alpha of 0.85 for Belgium and 0.9 for the Netherlands, with only four disagreements remaining across the data (out of a possible 160). The Krippendorff’s alpha levels were interpreted as satisfactory, and the remaining disagreements resolved via discussion among the wider research team.

Further, dual coding of the demographic data associated with 100 random ads using the screenshots collected by the initial coder (which are all publicly shared at the data deposit link) found that inter-rater reliability was very high with a product-moment correlation coefficient of 0.99 [60]. This process also allowed our data repository’s archiving quality to be spot-checked.

## Results

### Widespread Suspected Illegal Advertising

Among 372 unique games, 176 games (47.3%) advertised in either Belgium or the Netherlands, among which, 172 games (97.7%) advertised in Belgium, while 174 (98.9%) advertised in the Netherlands as shown in online supplementary material 1. In total, 1,574 suspected illegal ads for games with paid loot boxes and social casino games published in Belgium were identified.

These were viewed a total of at least 4.5 million times (*M* = 2,863, *SD* = 18,021). It must be noted that the “reach” data provided by Meta refer to the number of unique accounts that saw the ad at least once, meaning that multiple views of the same ad by the same account were not counted, although it was not publicly explained how long a user must view the ad (e.g., at least three seconds or 10% of a 30-second video) before an instance of “reach” is counted [61]. Accordingly, the total “impression” numbers for the ads would have been higher because the same account may have been shown the same ad multiple times [62]. In addition, many of the ads studied were part of then active advertising campaigns meaning that their impression numbers would have risen further after they were sampled. Some ads were studied almost immediately after they were first published (as we sampled the most recently published ads), thus resulting in (then) very low impression numbers having been recorded. Nonetheless, these data indicate that the advertising of video games containing illegal gambling-like video game mechanics was widespread and reached many people.

Table 1 shows the 10 games whose ads had the widest reach in Belgium in our sample. The one single most widely circulated ad was for *Harry Potter: Puzzles & Spells* and reached nearly 400,000 unique Belgian users. Belgium has a population of 11.8 million [63], meaning that 3.4% of the entire country saw that one single ad, thus demonstrating the incredible potential reach of social media advertising generally and of these suspected illegal ads specifically.

**Table 1.** Reach of ads published in Belgium by games known to contain loot boxes or social casino mechanics broken down by age range and gender

Game title	Reach by age range							Reach by gender			Overall	
	13–17	18–24	25–34	35–44	45–54	55–64	65+	Men	Women	Unknown	Total Belgian reach	Widest single ad reach
*Harry Potter: Puzzles & Spells*	0 (0.0%)	244,758 (31.7%)	279,013 (36.1%)	130,400 (16.9%)	70,379 (9.1%)	30,260 (3.9%)	17,638 (2.3%)	242,721 (31.4%)	523,544 (67.8%)	6,183 (0.8%)	772,448	396,699
*WWE 2K24*	0 (0.0%)	101,628 (15.5%)	169,661 (26.0%)	109,277 (16.7%)	76,017 (11.6%)	84,172 (12.9%)	113,036 (17.3%)	525,689 (80.4%)	124,160 (19.0%)	3,942 (0.6%)	653,791	220,059
*King’s Choice*	0 (0.0%)	265,057 (59.7%)	142,793 (32.1%)	26,567 (6.0%)	7,146 (1.6%)	1,681 (0.4%)	1,010 (0.2%)	49,907 (11.2%)	386,802 (87.1%)	7,545 (1.7%)	444,254	187,542
*Gossip Harbor: Merge & Story*	16 (0.0%)	191,016 (54.1%)	117,334 (33.2%)	30,281 (8.6%)	8,679 (2.5%)	2,149 (0.6%)	3,516 (1.0%)	35,419 (10.0%)	315,369 (89.3%)	2,203 (0.6%)	352,992	210,722
*NBA 2K24*	0 (0.0%)	102,743 (57.6%)	46,673 (26.2%)	17,209 (9.6%)	6,891 (3.9%)	2,209 (1.2%)	2,736 (1.5%)	139,714 (78.3%)	36,592 (20.5%)	2,155 (1.2%)	178,461	86,512
*The Ants: Underground Kingdom*	0 (0.0%)	37,242 (21.1%)	71,181 (40.3%)	45,241 (25.6%)	15,706 (8.9%)	5,029 (2.9%)	2,052 (1.2%)	143,126 (81.1%)	30,931 (17.5%)	2,394 (1.4%)	176,451	54,339
*The Elder Scrolls Online*	0 (0.0%)	24,144 (14.6%)	95,060 (57.4%)	33,289 (20.1%)	12,709 (7.7%)	565 (0.3%)	1 (0.0%)	154,320 (93.1%)	10,097 (6.1%)	1,351 (0.8%)	165,768	48,322
*World of Tanks*	0 (0.0%)	4,375 (2.9%)	53,657 (35.5%)	46,895 (31.0%)	44,947 (29.7%)	1,034 (0.7%)	323 (0.2%)	149,917 (99.1%)	1,184 (0.8%)	130 (0.1%)	151,231	47,032
*Puzzles & Survival*	0 (0.0%)	10,961 (8.8%)	43,842 (35.1%)	38,736 (31.0%)	21,897 (17.5%)	7,416 (5.9%)	2,244 (1.8%)	100,505 (80.3%)	22,449 (18.0%)	2,142 (1.7%)	125,096	46,176
*Royal Match*	0 (0.0%)	7,565 (7.9%)	27,342 (28.6%)	31,712 (33.1%)	17,113 (17.9%)	9,452 (9.9%)	2,503 (2.6%)	58,876 (61.5%)	35,941 (37.6%)	870 (0.9%)	95,687	29,468

Nearly all (91.5%) ads studied targeted all genders or did not specify a target gender; 6.9% targeted men only; and 1.6% targeted women only. However, in total, the ads reached 2.8 million men (62.0%), but only 1.7 million women (37.1%), in addition to 42,000 people of “unknown” genders (0.9%). Thus, overall, the ads were shown to 1.6 times more male users than female users.

However, at a more granular, individual game title level, this gender imbalance was not consistent. Perhaps due to game genre differences, much more extreme imbalances were observed, including also in the opposite direction. Among the 10 games whose ads had the widest reach in Belgium in our sample as shown in Table 1, except for *World of Tanks* (four of whose 10 ads targeted men only based on gender), all the other nine games did not target specific genders with any of their ads. Nonetheless, the reach of their ads to each gender was not balanced. *The Elder Scrolls Online* ads were delivered to 15.3 times more male users than female users, while *Gossip Harbor: Merge & Story* ads were delivered to 8.9 times more women than men. *World of Tanks* ads (likely partially because some of them targeted men only) were delivered to 126.6 times more male users than female users.

### Rare Instances of Potential Compliance Actions

Only four of all 174 games (2.3%) that advertised in the Netherlands were not advertised even once in Belgium. The specific countries that were chosen to be targeted by the ads of these four games were further scrutinized. Three games, including *Marvel Puzzle Quest*, simply did not include Belgium in its targeting and chose a few other countries (e.g., Austria and Finland only, alongside the Netherlands) to target as shown in Figure 1. The one other game, *Dragonheir: Silent Gods*, specifically excluded Belgium and Slovakia (which an official EU report had identified as then investigating whether paid loot boxes broke that country’s gambling law [64]) from targeting but included all other countries in the world as shown in Figure 2.

**Fig. 1.**
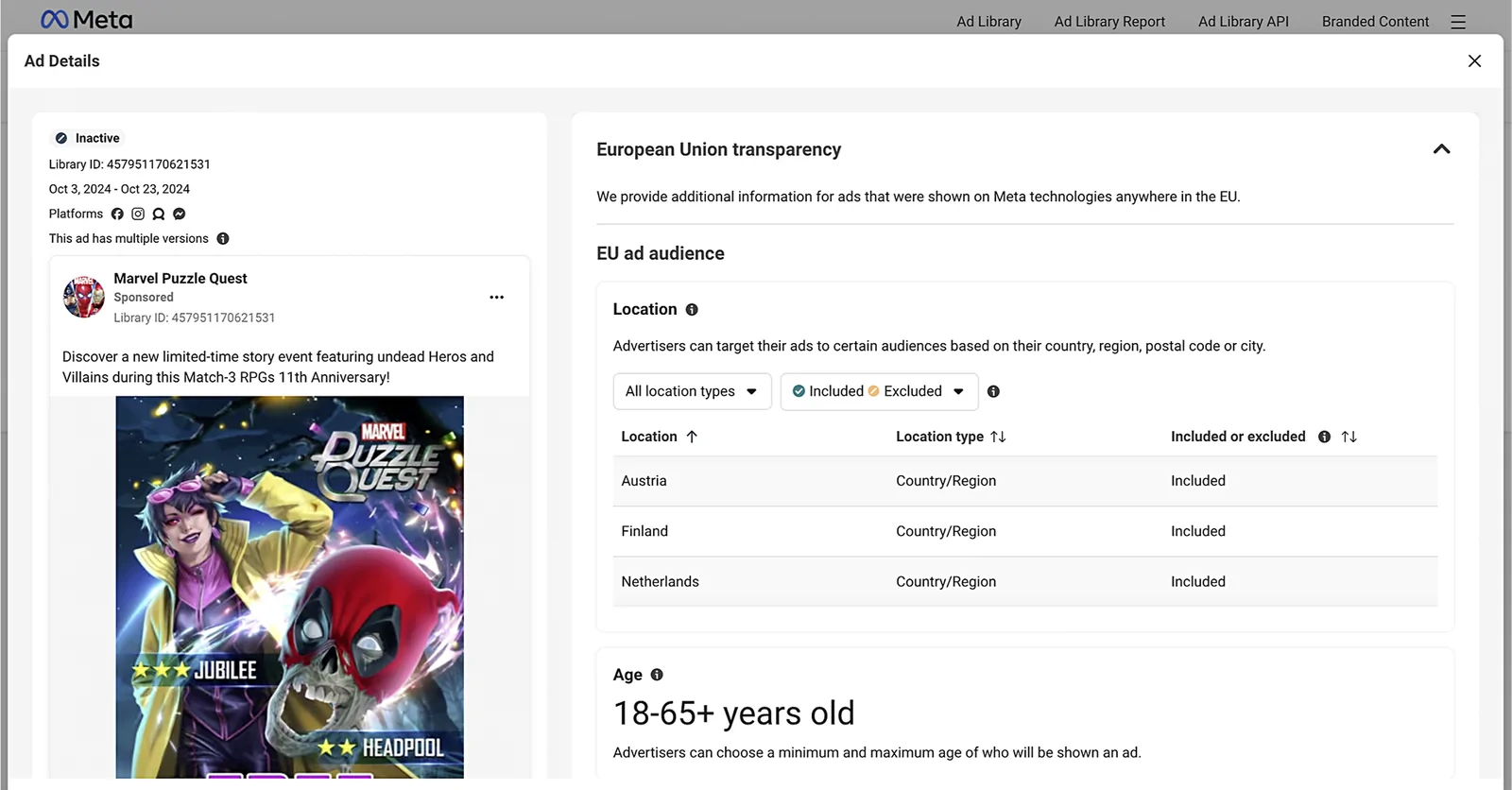
Meta ad ID# 457951170621531 for *Marvel Puzzle Quest* neither excluded nor included Belgium in its targeting but included just two other European countries in addition to the Netherlands, which is less indicative of deliberate compliance action. © 2024 Level Infinite.

**Fig. 2.**
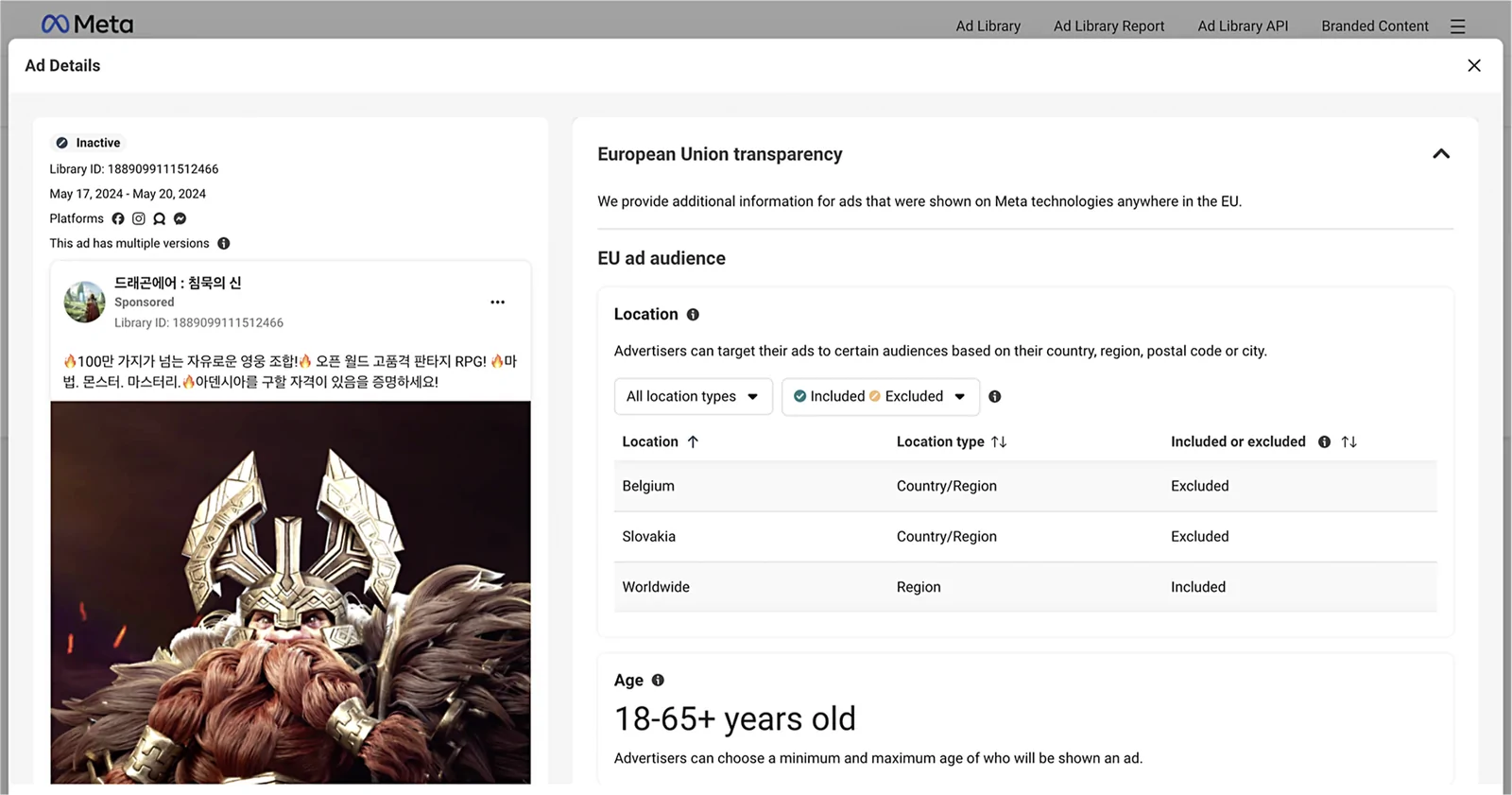
Meta ad ID# 1889099111512466 for *Dragonheir: Silent Gods* specifically excluded Belgium and Slovakia from targeting while including all other countries, which is indicative of deliberate compliance action. © 2024 Level Infinite.

Some individual ads of certain games, such as *Dragon Ball Z Dokkan Battle* – which is a game that is known to have previously taken compliance action to prevent Belgian users from purchasing paid loot boxes inside the game, although this could be easily circumvented [5] – targeted the whole Europe region but specifically excluded Belgium, as shown in Figure 3. However, inconsistently, other ads for the same game *did* target Belgium.

**Fig. 3.**
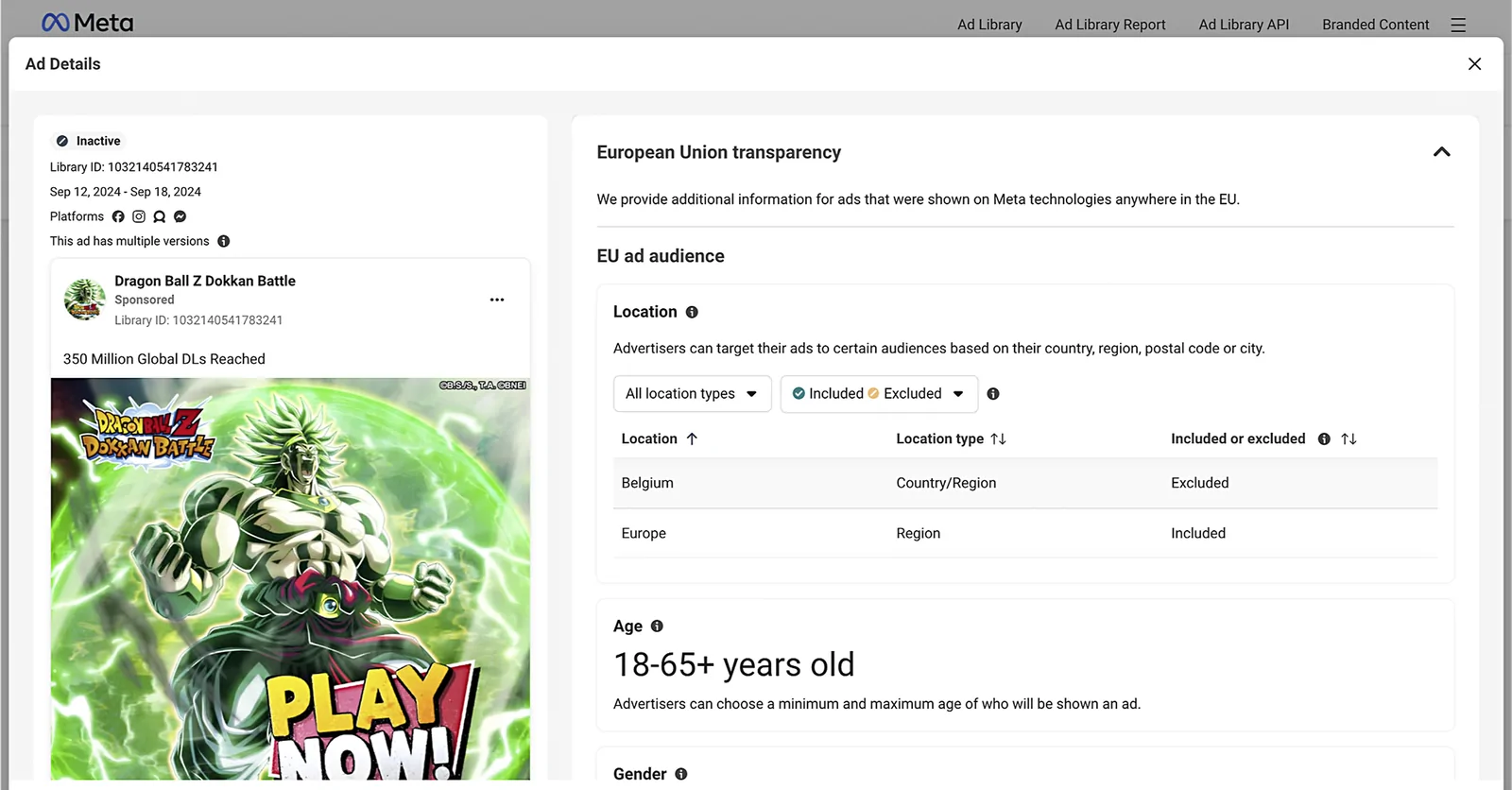
Meta ad ID# 1032140541783241 for *Dragon Ball Z Dokkan Battle* specifically excluded Belgium from targeting while including all of Europe, which is indicative of deliberate compliance action. © 2024 Akatsuki & Bandai Namco Entertainment.

*Governor of Poker 3*, a social casino game which also previously implemented technical measures inside the game to prevent Belgian users from spending money on illegal gambling (albeit also easily circumventable) [5], did advertise in Belgium but stopped doing so after March 2024, even though it continued to advertise in the Netherlands and most other European countries up to and including the time of writing (*i*.*e*., September 2026). The ads targeting most other European countries did not specifically exclude Belgium (i.e., list Belgium as a country that has been “excluded,” as in the examples above shown in Fig. 2 and Fig. 3) but simply did not include it (i.e., Belgium could not be found in the list of many countries that were “included”) as shown in Figure 4. As discussed below, these rare cases represent companies potentially having taken compliance action to respect Belgium’s ban on paid loot boxes and social casino games and their advertising.

**Fig. 4.**
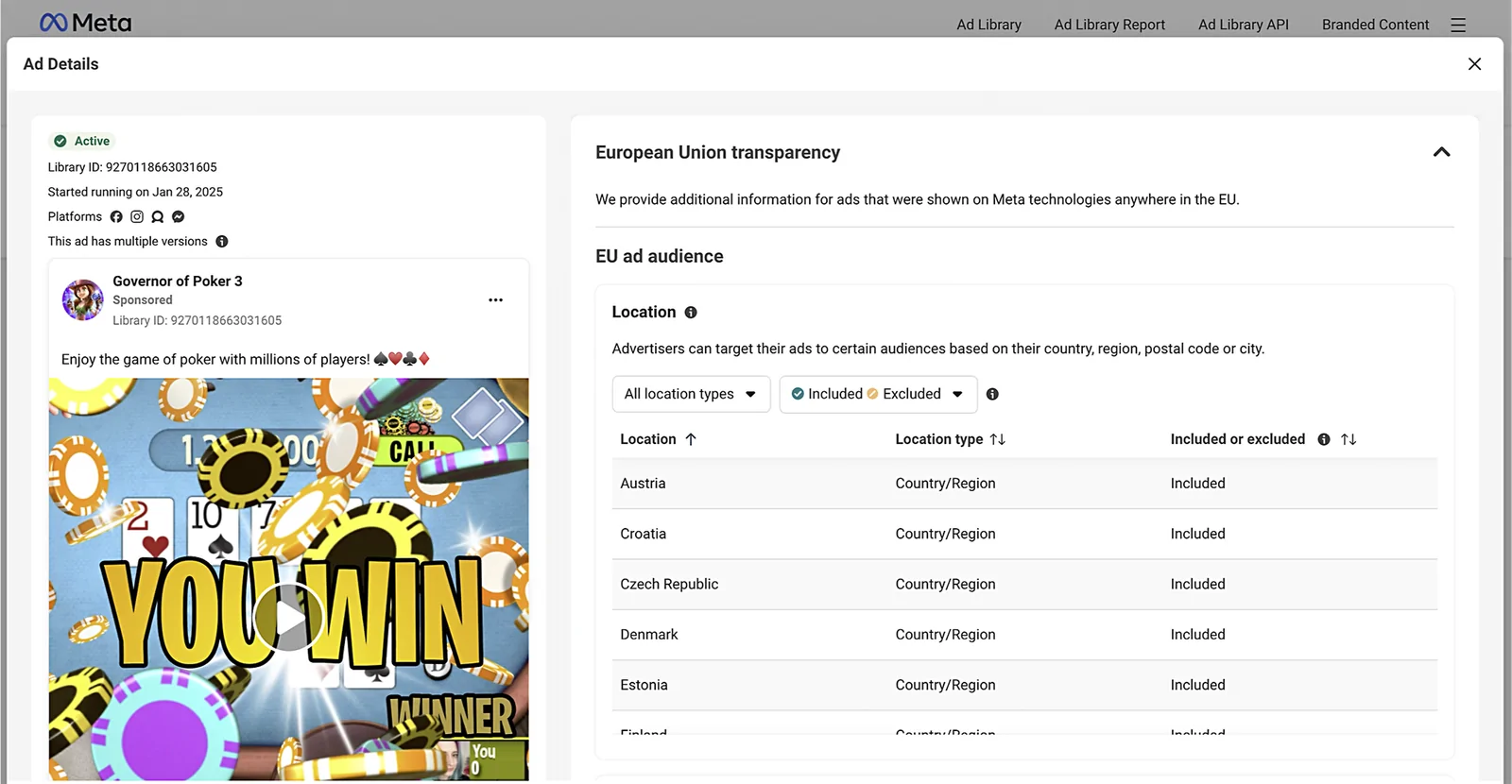
Meta ad ID# 9270118663031605 for *Governor of Poker 3* neither excluded nor included Belgium in its targeting but included most other European countries, which is also indicative of deliberate compliance action. © 2025 Playtika.

### Social Casino Subsample

Applying the definition of a social casino game as any game whose predominate feature is to allow the player to spend real-world money to simulate participating in traditional gambling activities, e.g., slot machines, poker, bingo, or craps, as adopted by previous paid loot box research [5], 36 of the 372 games (9.7%) were deemed to be social casino games. Among these, 26 games (72.2%) were advertised in Belgium. None of these were advertised only in the Netherlands but not in Belgium. We note, however, that the aforementioned *Governor of Poker 3* is a social casino game that likely took deliberate compliance action, but only more recently, as detailed above.

### Targeting and Reaching of Under-21s and Under-18s

As mentioned, young people aged under 21 years are not allowed to participate even in licensed forms of *legal* online gambling (except National Lottery products that are irrelevant for present purposes), and the criminal penalty for the crime of advertising illegal online gambling may be doubled if under-21s (i.e., more vulnerable people) are targeted. However, 1,141 of 1,574 suspected illegal ads (72.5%) were set to potentially target those aged under 21 years. Companies could have, but failed to, set a higher age target range.

We are unable to report the exact number of under-21s reached by these ads because the Meta Ad Library only provided ad reach data based on certain age brackets, and the 18–24 age bracket is too wide as it also partially includes people aged 21 years or above, but below 25 years. However, we can explore this as a hypothetical. If we assume that people belonging to each of the seven different years of age within that bracket were equally likely to have been reached because, approximately, there would be a similar number of people belonging to each age in the national population, then three-seventh of all 18–24 reach could be presumed to have been toward 18–20-year-olds (i.e., under-21s) specifically. The combined reach of the 1,141 suspected doubly illegal ads that targeted under-21s–for the 18–24 age bracket specifically–was 1.27 million or 28.2% of the total reach of all age groups. We therefore estimate that conceivably, over 544,000 reach or 12.1% of all reach was toward under-21s. While targeting people aged 21 years or above with illegal online gambling advertising is already a crime, the targeting of under-21s is an aggravating factor.

## Discussion

Many popular games with paid loot boxes and gacha mechanics (e.g., *Clash Royale* and *Genshin Impact*) and social casino games (e.g., *Coin Master* and *Monopoly Go!*), both of which are illegal in Belgium because they contravene gambling law, are suspected to have been widely unlawfully advertised to Belgian social media users. Even our relatively small sample of 1,574 ads (in the context that tens of thousands of such suspected illegal ads were likely published in Belgium every year in the past decade) were viewed over 4.5 million times, including conceivably 544,000 times by young people aged under 21 years (estimated based on our hypothetical assumptions), who are known to be more vulnerable to gambling harms [65, 66] and thus not permitted to participate even in most licensed and regulated forms of gambling in Belgium.

We identified rare cases of potential compliance action: specifically, either (i) excluding Belgium from targeting or (ii) choosing not to include Belgium in the list of countries that are targeted. However, importantly, games may have not been advertised for a variety of reasons, including that the game was no longer operational or that the company simply decided not to spend money promoting that game. Therefore, not having been advertised in Belgium alone is not necessarily indicative of a company taking compliance action. Instead, what is more suggestive of taking compliance action is if a game was advertised in the Netherlands (thus showing that the game was operational and that the company was willing to invest financial resources into promoting it, at least in another country) but was not advertised in Belgium. Only four of all 174 games (2.3%) that advertised in the Netherlands belonged to this category. We also identified how at least one game, *Dragon Ball Z Dokkan Battle*, excluded Belgium from the targeting of certain ads but also ran other ads that included Belgium as a target country. Furthermore, we found at least one game, *Governor of Poker 3*, that appears to have stopped advertising in Belgium more recently even though it did advertise to Belgian users previously.

Our findings are unsurprising in the paid loot box regulation context: previous research has identified how the most popular games continued to illegally sell paid loot boxes in Belgium despite the ban [5]; how the advertising of games with paid loot boxes generally failed to disclose paid loot box presence as required in the UK and South Korea [37, 38]; and how video games failed to comply with many aspects of Dutch consumer law, such as the requirement to disclose a price in euros for all in-game purchases, in addition to the price in fictional virtual currencies [6]. Our findings contribute to the evidence that the highly restrictive and arguably practically unenforceable blank ban approach to paid loot box regulation applied in Belgium is not effective and helpful at reducing harm when the infrastructure that is required to monitor and uphold the law is not properly funded. Consequently, cheaper, quicker, and more informal enforcement options should be considered. One easily actionable recommendation for the Belgian gambling regulator is to publish a guide on how video game companies can ensure their advertising excludes Belgian users from its potential audience, e.g., screenshots showing how to set exclusion options based on location on various popular advertising platforms, such as Meta’s Ads Manager, as shown in Figure 5. This guide should then be widely circulated to video game companies, potentially through app stores and via industry trade bodies, like Video Games Europe.

**Fig. 5.**
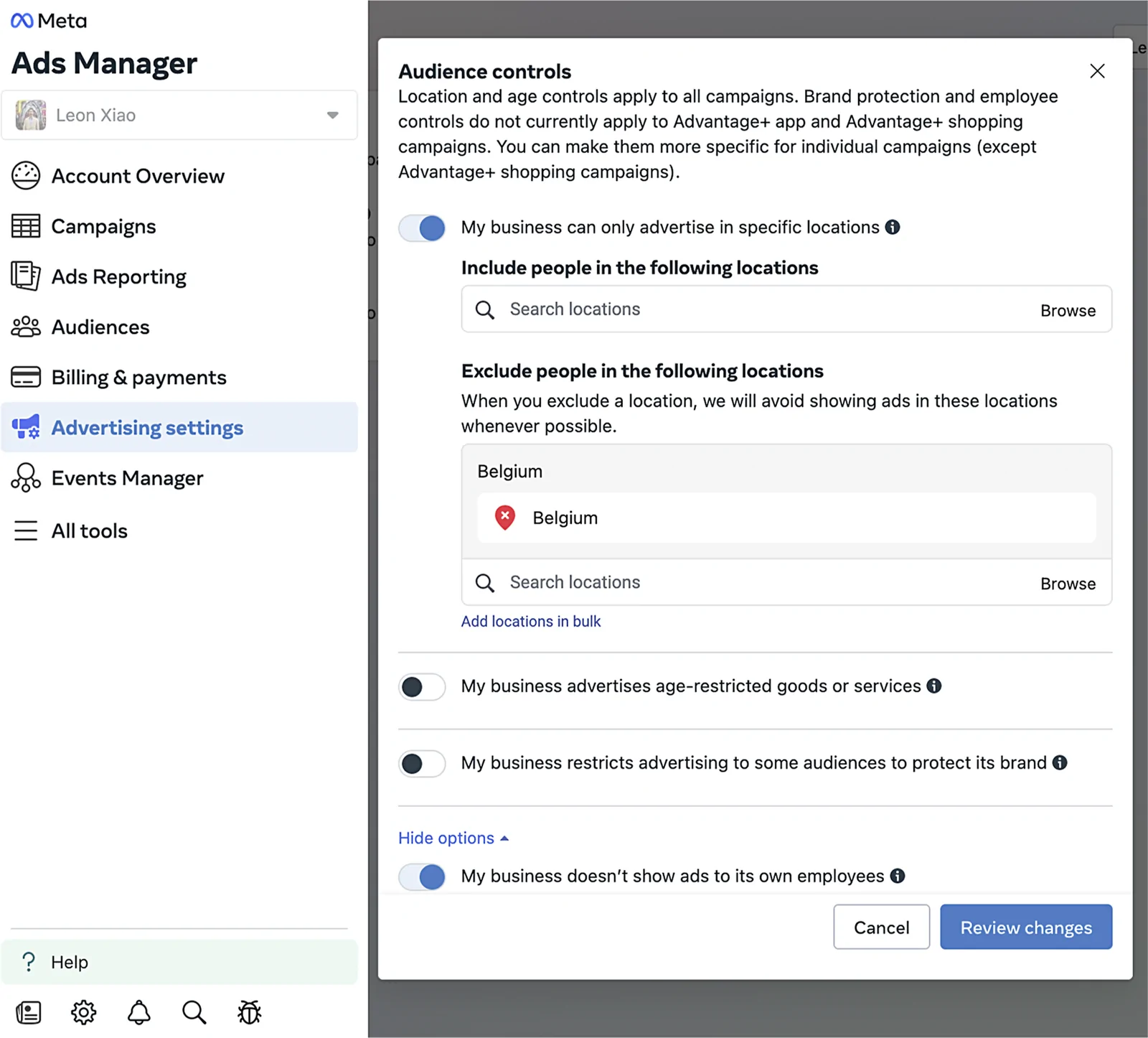
A screenshot showing how to exclude Belgium from targeting using Meta’s Ads Manager. © 2025 Meta.

The regulator should also demand Meta to immediately remove all ads suspected to be advertising illegal gambling from being shown in Belgium and restrict the relevant accounts (which are nearly all dedicated to advertising a specific game containing illegal gambling) from showing any ads in Belgium as a precautionary measure. The costs of content moderation and enforcement should be borne by Meta, as an advertising platform that takes a fee from the advertiser and thus profits from each and every one of these suspected illegal adverts. Recently, a Belgian player has sued Apple seeking damages for harms caused by paid loot boxes because the Apple App Store hosted illegal content (i.e., a game with illegal paid loot boxes) [44, 45]. That case settled out of court, and so there will be no longer be a Court of Justice of the European Union (CJEU) judgment deciding whether Apple could be held liable given its level of knowledge of the presence of illegal paid loot boxes (i.e., perhaps lacking specific knowledge of that particular game’s illegality, but knowing or should have known the widely publicized Belgian regulatory position that paid loot boxes are illegal gambling). To prevent social media companies from relying on that same potential defense to avoid taking legal responsibility, Meta and other platforms should be put on notice and given specific knowledge that they are hosting suspected illegal advertising of games with paid loot boxes and social casino games. After they have been made aware of those specific pieces of suspected illegal content, they could then be held legally liable unless they promptly remove said content.

Apple and Google should also be given specific notice of their hosting of illegal games with paid loot boxes and social casino games on app stores by the regulator to remove their potential immunity as intermediaries that host information. Indeed, these platforms (both social media and app stores) hold significant power over the availability and visibility of either ads or games through their content moderation process. They also profit considerably from making these ads and games available to consumers. In practice, due to funding constraints on official government gambling and consumer regulators [49], these platforms are likely the most cost-effective alternatives for enforcing video game regulation based on the divergent rules of different jurisdictions (e.g., the ban on paid loot boxes and the advertising of games containing them in Belgium). These platforms should be held accountable by regulators for ensuring the legality of any content that they make available to users, which is cheaper and easier than the regulator itself attempting to enforce the law against individual video game companies based in many different foreign countries. These platforms’ content moderation practices must be improved to ensure that illegal content cannot be easily published. If any is unfortunately published, then a robust complaint-handling process should be in place to urgently remove it upon receipt of legitimate concerns as is required by Articles 9 and 20 of the DSA, for example.

The present research provides a method that can be used not only for academic research but also by regulators for compliance monitoring and enforcement purposes. Article 39 of the DSA placed an obligation on social media platforms to provide advertising repositories to facilitate better public (including academic) and regulatory scrutiny of illegal advertising and counter potential risks to public health per Recital 95. Accordingly, the data access that the DSA has enabled should be fully utilized by all stakeholders to advance the public good. The EU should require platforms to provide even more detailed data: for example, instead of permitting ad reach data to be inexactly provided based on age brackets only (e.g., people between 18 and 24), demand that platforms provide precise breakdowns by each individual year of age (e.g., people aged 18 specifically). The lack of this specific detail hindered the present research and rendered its findings less accurate. Other countries beyond the EU should recognize the obvious benefits of these legal provisions and promptly require the same repository and data be provided in their country to better protect their citizens.

Indeed, social media advertising repositories present many exciting research opportunities. Previous research has suggested, based on commonly held belief, that certain games’ advertising targets specific genders [67]. This can now be proven. Nearly all ads (91.5%) targeted all genders or did not target, while 6.9% targeted men only and 1.6% targeted women only. Accordingly, even though there were four times as many ads targeting men only as compared to ads targeting women only, there was no significant bias toward men being targeted overall. However, overall, the ads unequally reached 2.8 million men (62.0%) but only 1.7 million women (37.1%), in addition to 42,000 people of “unknown” genders (0.9%). This appears to suggest a potential “algorithmic bias” [68] toward showing video game ads to men, even when they were not being specifically targeted by those ads. However, the imbalances were even more extreme and could have been in either direction when viewed from a more granular, individual game title perspective: a PC- and console-based fantasy role-playing game’s ads (*The Elder Scrolls Online*) were delivered to 15.3 times more men, while a “casual” mobile puzzle game’s ads (*Gossip Harbor: Merge & Story*) were delivered to 8.9 times more women, even though neither game’s ads technically targeted users of a specific gender. The algorithm appears to have some other undisclosed methods for determining which user should be shown a specific ad. This might be entirely innocent and even beneficial for both advertisers and users: if men are more interested in the video games being advertised than women, then, consequently, users who are more likely to use the products being advertised are being connected with them more often (although we note in this particular instance, illegal products that should not have been shown to anyone were being advertised).

Conversely, previous research on paid loot boxes [15], social casinos [69], and traditional gambling [70] found that men may be more vulnerable to harm (although recognizing that other demographics, like older women, also have unique vulnerabilities with social casinos in particular [28, 71, 72]). We found that men are also more often exposed to the advertising of these potentially harmful products. Whether this was caused by the intentional targeting of the male gender by advertisers or by algorithmic bias in favor of serving these ads to men, harm might have consequently been exacerbated through social media advertising. Future research should study when and why certain demographics are targeted and reached (e.g., in our sample, rather stereotypically, match-3 games themed around fashion and interior design targeted and reached women, while strategy games themed around war targeted and reached men) and whether potentially vulnerable demographics (e.g., based on age) are targeted and reached. To answer those questions, an analysis of the advertising repositories would need to be supplemented with, e.g., game analysis, interviewing advertisers, and ethnographic research observing how users engage with these ads in practice [73].

## Limitations

As to limitations, the data provided by the Meta ad repository are not independently verifiable. However, we are confident in assuming their reasonable accuracy and reliability (except as to the reach data toward under-18s due to many children using false adult accounts [74]) because they are being provided to satisfy DSA legal obligations. Any failure on the part of Meta to provide correct data could be severely sanctioned by the European Commission with heavy fines. We collected the data for each game and each ad at a specific time. If the data were collected at a different time, we may have obtained different results because the most recently published ads may have changed, and active advertising campaigns would have gained more impressions.

The data collection was based on games that are known to contain paid loot boxes and/or are social casino games from previous research. It is conceivable that a few of those games subsequently removed the relevant mechanics and therefore no longer contained paid loot boxes and/or constituted social casino games following software updates, so their advertising would then become lawful. However, previous research has established that very few games would fall within that category [5], and players can also circumvent technical measures meant to prevent them from purchasing paid loot boxes in Belgium [47].

We cannot conclusively say any game intentionally complied with the requirement not to advertise illegal gambling in the form of video games with paid loot boxes and social casino games in Belgium. We found strong circumstantial evidence to suggest deliberate compliance actions were taken by a few companies, but a few more companies may have taken such actions (in particular, games that did initially advertise but stopped doing so more recently and those that may have inconsistently complied with the law with some ads but not others). However, the headline finding that video games containing paid loot boxes and social casino games are suspected to have been widely and illegally advertised in Belgium by many companies is uncontroversial: many suspected illegal current and historical ads for many popular games were found to have been published in the country.

We did not conduct content analysis on the ads’ graphic materials. Doing so through future research may reveal that some ads highlighted the paid loot boxes or social casino mechanics of a game specifically, while other ads featured other aspects of gameplay only. The former would arguably be more culpable and certainly illegal as illegal gambling products were more directly advertised, while the latter were opaquer about the presence of gambling-like mechanics in those games, which players might not notice until they buy or download and then start playing the game [37–39].

## Conclusion

Many popular games with paid loot boxes and social casino games, both of which constitute illegal gambling in Belgium, were widely – and suspected to have been illegally – advertised to Belgian social media users. The 1,574 suspected illegal ads studied, which represent only a portion of all suspected illegal video game ads that would have been published in Belgium, were viewed over 4.5 million times, including conceivably 544,000 times by under-21s (estimated based on our hypothetical assumptions), who are not legally permitted to gamble online in Belgium. Companies have not only failed to remove illegal gambling-like video game mechanics from the Belgian market [5] but are paying money to actively attract more Belgian users to engage with these illegal products. Large technology companies, like Meta, Apple, and Google, should be held accountable for conducting proper content moderation and ensuring illegal contents (both in the form of video games containing illegal gambling and ads for those games) are not easily published and can be removed quickly upon receipt of any complaints. The data access enabled by the EU Digital Services Act can help the public (including academic researchers) and regulators better scrutinize potentially harmful and illegal commercial practices and potentially enforce against them. Other countries should impose similar data provision obligations on technology companies to ensure local research could be conducted.

## Acknowledgments

Thanks to Callum Deery for further analyzing the reach data during revision. L.Y.X. plays and enjoys video games and broadly views the activity very positively, except for certain aspects (e.g., monetization) that he believes should be subject to more scrutiny. In terms of L.Y.X.’s personal engagement with loot boxes, he has played and continues to play video games containing loot boxes, such as *Hearthstone* (Blizzard Entertainment, 2014) until 2018 and *Genshin Impact* (miHoYo, 2020) and *Zenless Zone Zero* (miHoYo, 2024) since their initial release until early 2025. He therefore engaged with nonpaid loot boxes on a regular basis. However, he has never purchased any loot boxes with real-world money aside from negligible spending for research purposes to, e.g., confirm the presence of paid loot boxes. E.P. has previously played games intensively, between the ages of 16–18, including *Hearthstone,* which includes loot boxes, and spent a small amount of money on loot boxes in this game. She quickly realized she found this unrewarding and never spent money on a game again. She no longer plays any video games (outside of what is required for research).

## Statement of Ethics

An ethics statement was not required for this study type since no human or animal subjects or materials were used.

## Conflict of Interest Statement

L.Y.X. has provided paid consultancy and research services for (1) Public Group International Ltd (t/a PUBLIC) (Companies House No. 10608507), commissioned by the UK Department for Culture, Media and Sport (DCMS) to conduct independent research on understanding player experiences of loot box protections (October 2024–May 2025); (2) the Council of Europe International Cooperation Group on Drugs and Addiction (the Pompidou Group) on a project concerning the risks of online gambling and gaming to young people co-funded by the European Union via the Technical Support Instrument and implemented by the Council of Europe, in cooperation with the European Commission (December 2024–May 2025); and (3) the Institute of Public Health on a report concerning the advertising of harmful products to children funded by the Irish Department of Health and intended for its Online Health Taskforce (July 2025). L.Y.X. was supported by a PhD Fellowship funded by the IT University of Copenhagen (IT-Universitetet i København) (December 2021–November 2024). L.Y.X. was employed by LiveMe, then a subsidiary of Cheetah Mobile (NYSE: CMCM), as an in-house counsel intern from July to August 2019 in Beijing, China. L.Y.X. was not involved with the monetization of video games by Cheetah Mobile or its subsidiaries. L.Y.X. undertook a brief period of voluntary work experience at Wiggin LLP (Solicitors Regulation Authority No. 420659) in London, England, in August 2022. L.Y.X. has contributed to research projects enabled by data access provided by the video game industry, specifically Unity Technologies (NYSE:U) (October 2022–August 2023). L.Y.X. has been invited to provide advice to the UK Department for Digital, Culture, Media and Sport and its successor (the Department for Culture, Media and Sport [DCMS]) on the technical working group for loot boxes and the Video Games Research Framework. L.Y.X. was the (co-)recipient of three Academic Forum for the Study of Gambling (AFSG) postgraduate research support grants (March 2022, January 2023, and July 2024) and a minor exploratory research grant (May 2024) derived from “regulatory settlements applied for socially responsible purposes” received by the UK Gambling Commission and administered by Gambling Research Exchange Ontario (GREO) and its successor (Greo Evidence Insights; Greo). L.Y.X. accepted funding to publish open-access academic papers from GREO and the AFSG that was received by the UK Gambling Commission as above (October, November, and December 2022, November 2023, and May 2024). L.Y.X. was the recipient of an Elite Research Travel Grant 2024 (EliteForsk-rejsestipendium 2024) awarded by the Agency for Higher Education and Science of the Danish Ministry of Higher Education and Science (Uddannelses-og Forskningsstyrelsen under Uddannelses-og Forskningsministeriet) (February 2024). L.Y.X. has accepted conference travel and attendance grants from the Socio-Legal Studies Association (February 2022 and February 2023); the Current Advances in Gambling Research Conference Organising Committee with support from GREO (February 2022); the International Relations Office of The Jagiellonian University (Uniwersytet Jagielloński), the Polish National Agency for Academic Exchange (Narodowa Agencja Wymiany Akademickiej [NAWA]), and the Republic of Poland (Rzeczpospolita Polska) with cofinancing from the European Social Fund of the European Commission of the European Union under the Knowledge Education Development Operational Programme (May 2022); the Society for the Study of Addiction (November 2022, March 2023, and November 2024); the organizers of the 13th Nordic SNSUS (Stiftelsen Nordiska Sällskapet för Upplysning om Spelberoende; the Nordic Society Foundation for Information about Problem Gambling) Conference, which received gambling industry sponsorship (January 2023); the MiSK Foundation (Prince Mohammed bin Salman bin Abdulaziz Foundation) (November 2023); the UK Gambling Commission (March 2024); the Video Game and Art Research Center, Peking University (April 2026); and the Alberta Gambling Research Institute (May 2026). L.Y.X. has received honoraria from the Center for Ludomani for contributing parent guides about mobile games for Tjekspillet.dk, which was funded by the Danish Ministry of Health’s gambling addiction pool (Sundhedsministeriets Ludomanipulje) (March and December 2023), the Fundació Pública Tecnocampus Mataró-Maresme (TecnoCampus Mataró-Maresme Foundation) for a guest lecture (November 2023), the Young Men’s Christian Association (YMCA) of Greater Toronto Youth Gambling Awareness Program for a presentation, which was funded by the Government of ON, Canada (March 2024), and Lunds universitet (Lund University) for the right to translate parent guides about mobile games into Swedish for Kollaspelet.se, which was funded by Mediemyndigheten (the Swedish Agency for the Media) and Barnahus Stockholm (December 2024); Shenkar College of Engineering, Design and Art for a guest lecture (December 2024); and DiGRA Korea and the Game-n-Science Institute (게임과학연구원) under the Game Culture Foundation (게임문화재단) under the Ministry of Culture, Sports and Tourism of South Korea (문화체육관광부) for participating in an academic research survey (January 2025). L.Y.X. received royalties by virtue of the copyright subsisting in some of his publications from the Authors’ Licensing and Collecting Society (ALCS) (Companies House No. 01310636) (March 2023, 2024, 2025, and 2026). A full gifts and hospitality register-equivalent for L.Y.X. is available at https://www.leonxiao.com/about/gifts-and-hospitality-register. The up-to-date version of L.Y.X.’s conflict of interest statement is available at https://www.leonxiao.com/about/conflict-of-interest. E.P. is the recipient of an Academic Forum for the Study of Gambling (AFSG) Postgraduate Research Support Grants that were derived from “regulatory settlements applied for socially responsible purposes” received by the UK Gambling Commission and administered by Gambling Research Exchange Ontario (GREO) (March 2022). E.P. is currently employed on a project funded by GREO and has an honorary contract with the National Health Service (NHS) England. E.P. currently provides paid consultancy for Public Group International Ltd, commissioned by the UK Department for Culture, Media and Sport (DCMS) as part of conducting independent research on understanding player experiences of loot box protections, since October 2024. N.K. was employed with research funding from the AFSG. Funding for the AFSG is administered by Greo, for which the funds are sourced from regulatory settlements levied by the UK Gambling Commission. M.D. declares no conflict of interest. A.R. is Co-Chair of the Executive Committee of the Academic Forum for the Study Gambling (AFSG). Funding for the AFSG is derived from regulatory settlements for socially responsible purposes that are approved by the Gambling Commission and is administered by Greo Evidence Insights (Greo). A.R. has received funding from the Society for the Study of Addiction (SSA), Greo, National Institute of Health Research (NIHR), Lincolnshire County Council, Ashfield District Council, Health and Care Research Wales, Public Health Lincoln and Santander. She does not have any potential conflicts of interest in relation to gambling or the gambling industry.

## Funding Sources

This study was funded by an Academic Forum for the Study of Gambling (AFSG) Minor Exploratory Grant awarded to L.Y.X. and E.P. that was derived from “regulatory settlements applied for socially responsible purposes” received by the UK Gambling Commission and administered by Greo Evidence Insights (Greo) (May 2024). L.Y.X. is supported by a Presidential Assistant Professors Scheme Start-Up Research Grant awarded by the City University of Hong Kong (香港城市大學) (March 2025). Until November 2024, L.Y.X. was supported by a PhD Fellowship funded by the IT University of Copenhagen (IT-Universitetet i København), which was publicly funded by the Kingdom of Denmark. The funders had no role in the design, data collection, data analysis, and reporting of this study.

## Author Contributions

L.Y.X.: conceptualization, data curation, formal analysis, funding acquisition, methodology, project administration, resources, software, supervision, validation, visualization, and writing – original draft, review, and editing; E.P.: conceptualization, funding acquisition, methodology, resources, supervision, and writing – review and editing; N.K.: investigation, methodology, visualization, and writing – review and editing; M.D.: funding acquisition, validation, and writing – review and editing; A.R.: funding acquisition, resources, and writing – review and editing.

## Data Availability

All advertising materials analyzed, the raw data, and the analysis script and output are publicly available in the Open Science Framework at https://doi.org/10.17605/OSF.IO/2URYM.
